# Immunomodulatory effects of thalidomide in an experimental brain death liver donor model

**DOI:** 10.1038/s41598-021-98538-z

**Published:** 2021-09-28

**Authors:** Alexandre Chagas Santana, Wellington Andraus, Filipe Miranda Oliveira Silva, Humberto Dellê, Rafael Pepineli, Edvaldo Leal de Moraes, Cristoforo Scavone, Larissa de Sá Lima, Sabrina Degaspari, Sergio Brasil, Davi Jorge Fontoura Solla, Liliane Moreira Ruiz, Karina Andrighetti de Oliveira-Braga, Natalia Aparecida Nepomuceno, Paulo Manuel Pêgo-Fernandes, Stefan Gunther Tullius, Eberval Gadelha Figueiredo

**Affiliations:** 1 Neurological Surgery Department, University of Sao Paulo School of Medicine, Av. Dr. Enéas Carvalho de Aguiar, 255, 5th Floor, São Paulo, CEP: 05402–000 Brazil; 2 Gastroenterology Department, School of Medicine, University of Sao Paulo, Sao Paulo, Brazil; 3 Medical Science Department, Nove de Julho University, São Paulo, Brazil; 4 Organ Procurement Organization Department, School of Medicine, University of Sao Paulo, Sao Paulo, Brazil; 5 Molecular Neuropharmacology Laboratory, Department of Pharmacology, Institute of Biomedical Science, University of Sao Paulo, Sao Paulo, Brazil; 6 Cardiopneumology Department, School of Medicine, University of Sao Paulo, Sao Paulo, Brazil; 7 Department of Surgery, Division of Transplant Surgery, Brigham and Women’s Hospital, Harvard Medical School, Boston, MA USA

**Keywords:** Cytokines, Transplant immunology

## Abstract

Brain death is characterized by a generalized inflammatory response that results in multiorgan damage. This process is mainly mediated through cytokines, which amplify graft immunogenicity. We investigated the immunological response in a brain death liver donor model and analysed the effects of thalidomide, a drug with powerful immunomodulatory properties. Brain death was induced in male Lewis rats. We studied three groups: Control (sham-operated rats in which trepanation was performed without inserting the balloon catheter), BD (rats subjected to brain death by increasing intracranial pressure) and BD + Thalid (BD rats receiving thalidomide after brain death). After 6 h, serum levels of AST, ALT, LDH, and ALP as well as systemic and hepatic levels of TNF-α, IL1-β, IL-6, and IL-10 were analysed. We also determined the mRNA expression of MHC Class I and Class II, NF-κB, and macrophage infiltration. NF-κB was also examined by electrophoretic mobility shift assay. Thalidomide treatment significantly reduced serum levels of hepatic enzymes and TNF-α, IL-1-β, and IL-6. These cytokines were evaluated at either the mRNA expression or protein level in liver tissue. In addition, thalidomide administration resulted in a significant reduction in macrophages, MHC Class I and Class II, and NF-κB activation. This study reveals that thalidomide significantly inhibited the immunologic response and graft immunogenicity, possibly through suppression of NF-κB activation.

## Introduction

Over the past 50 years, liver transplantation has advanced dramatically and is considered the definitive therapy for end-stage liver failure. The number of people receiving liver transplants has increased annually worldwide. In 2019, in the United States, more than 8300 Americans underwent liver transplantation, whereas over 2000 liver transplantations were performed from deceased donors in Brazil^1^. Nevertheless, despite the substantial increase in the number of donors, donation rates are not growing as quickly as the demand for organs^1^. As a result, many patients die yearly waiting for a liver transplant. In addition, the COVID-19 pandemic outbreak is closely associated with high mortality in solid organ transplant recipients^2^.

A vast majority of the livers used for transplantation are obtained from brain-dead donors. Brain death (BD) is characterized by blood–brain barrier dysfunction, resulting in intense cellular and molecular activation that quickly evokes an immune response with both cellular and humoral components, both systemically and intragraftly, thereby increasing graft immunogenicity^3,4^. Therefore, BD itself has been recognized as an important risk factor for graft failure^5–8^.

These changes have been shown to result in a variety of complications that adversely affect hepatocyte function and therefore significantly decrease the viability of transplanted livers^9^. Although knowledge regarding BD has broadened during the past few years, the signals and mechanisms involved in the augmented immunogenicity of organs after BD remain obscure. Several investigations have postulated that immunomodulatory factors produced during BD may be mediated by increased levels of cytokines, such as interleukin TNF-α, IL-1β, and IL-6^6,10^.

In particular, the increase in these cytokines results in augmented inflammation and consequently increases the expression of major histocompatibility complex (MHC) and adhesion molecules, which amplify their immunogenicity in donor cells^4^. In this context, nuclear factor kappa B (NF-κB) has a remarkable role in coordinating the expression of a variety of genes, especially those that participate in inflammation and alloimmunity^11,12^.

NF-κB is a DNA-binding factor that promotes the expression of over 150 target genes. The majority of proteins encoded by NF-κB activation target genes that participate in the immune response, including TNF-α, IL-1β, and IL-6^12^. The activation of NF-κB has been confirmed in hepatocytes during inflammatory responses in human and experimental models^12–15^. Thus, intervention therapies that aim to inhibit proinflammatory cytokines and NF-κB activation represent potential approaches that could be beneficial for organs from brain-dead donors.

In this setting, the therapeutic application of thalidomide may represent an interesting strategy. As an immunomodulatory agent, thalidomide has been recognized as a suppressor of the production of cytokines, including TNF-α, IL-1β, and IL-6^16,17^. Additionally, thalidomide may also inhibit the expression of NF-κB through the degradation of IκB kinase activity when induced by proinflammatory cytokines^18^. To the best of our knowledge, no studies have analysed the role of thalidomide in this scenario.

The aim of this study was to analyse the potential beneficial effects of thalidomide in an experimental model of BD based on its anti-inflammatory and immunomodulatory properties.

## Materials and methods

### Animals

Inbred male Lewis (LEW) rats (9 to 10 weeks age) weighing approximately 250 to 300 g were studied. Animals were housed in a 22 °C room with a 12-h light/dark cycle and allowed ad libitum access to food and water before and after the surgical procedure. All experimental procedures were approved by the Institutional Ethical Research Board of the University of Sao Paulo, Brazil (permit number 031/17). All animals received care in accordance with international standards of animal care and experimentation in compliance with the ARRIVE guidelines.

### Brain death model

BD induction was performed using techniques described previously^19^. Briefly, rats were anaesthetized with isoflurane, intubated via tracheostomy, and then ventilated (MR-compatible Small Animal Ventilator. SA Instrument, Inc. NY. USA). Through frontolateral trepanation, a Fogarty-4F balloon catheter (Edwards Lifescience LLC, Irvine, CA, USA) was introduced into the intracranial cavity with the tip pointing caudally. The balloon was slowly inflated with 0.5 mL saline solution using a syringe pump. Thus, an increase in intracranial pressure induced progressive brain injury leading to BD. BD was clinically diagnosed by the occurrence of an autonomic storm and a test of brain stem reflexes, including the absence of corneal reflexes and dilated and fixed pupils, followed by an apnoea test.

### Ultrasonography assessments

**Figure 1:**
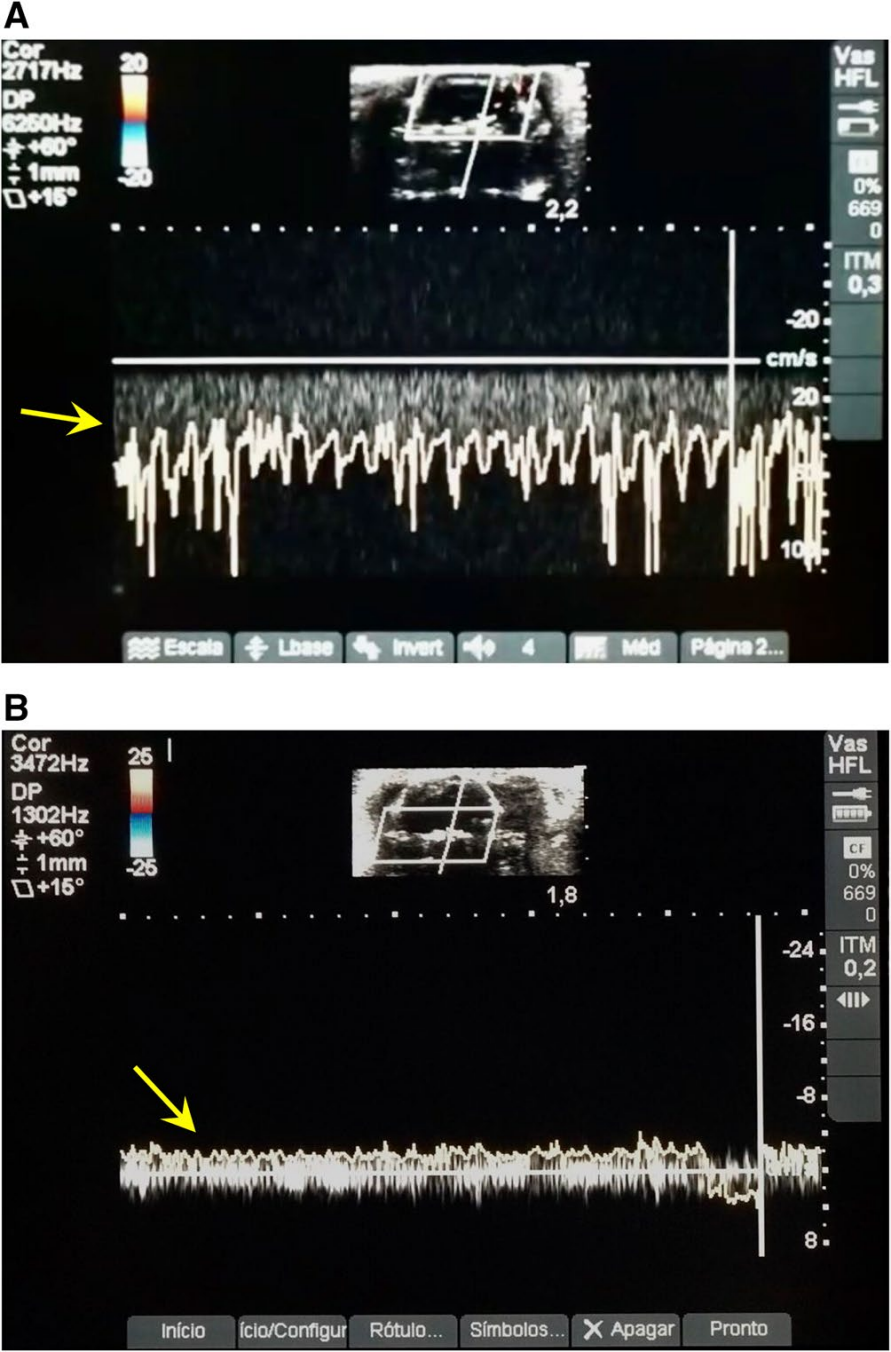
Ultrasound imaging and flow changes of the cerebral arteries measured in a cross-sectional direction. (**A**) Ultrasound imaging assessments before brain death induction. Note the presence of blood flow in the reverse direction (arrow). (**B**) Ultrasound imaging assessments 6 h after brain death induction. Note the blood flow compatible with brain blood circulatory collapse (arrow).

To confirm the diagnosis of BD, transcranial colour-coded sonography (TCCS) assessments were performed with Micromaxx (Sonosite, USA) ultrasound in conjunction with a 6 to 14 MHz linear transducer. Ultrasound conduction gel was applied to improve the conductivity. First, 2-D ultrasound imaging was used to visualize a cross-sectional B-mode image of the skull and brain structures of the animal. A colour Doppler ultrasound was then used, and the extra and intracranial arteries were indicated on the screen by two different colours (blue and red, where the blue colour indicates the blood flow away from the transducer and the red colour indicates the flow towards the transducer). The probe was placed above the skull, and the cross-sectional scan was performed by moving the probe from the back to the front. Imaging depth was set at 2 cm when applying zoom. Standardized settings were used for colour Doppler ultrasound: ultrasound frequency at 6.3 MHz, pulsed repetition frequency (PRF) at 4 kHz and frame rate of 65 frames/s were applied. A heating lamp was used to assure normothermia. The scalp was opened, and the first scan visualized the right ICA, right middle cerebral artery (MCA) and basilar artery (BA). Left ICA and MCA were not observed due to ICA cannulation. Doppler spectra were obtained in all vessels (Fig. 1A).

Following an initial examination, a burr hole was made with the aid of an electric drill; a catheter was introduced, and a balloon was inflated inside the skull, raising intracranial pressure (ICP). Systolic spikes under 10 cm/s were retrieved on the right ICA and basilar artery at the level of the skull base, compatible with brain blood circulatory collapse (Fig. 1B).

### Experimental groups and thalidomide treatment study design

To analyse the effects of thalidomide, three groups of rats (n = 8 per group) were included in this study: **Control** (sham-operated rats in which trepanation was performed without inserting the balloon catheter), **BD** (rats submitted to BD) and **BD + Thalid** (BD rats receiving thalidomide (200 mg/kg) by gavage immediately after BD). The dose of thalidomide was based on previous experimental studies^15,26,27^. After 6 h, liver samples were removed using sterile techniques and sectioned into 4 transverse sections. The sections were fixed in 4% buffered formaldehyde solution for immunohistochemistry analysis or stored at − 80 °C for real-time RT–PCR, multiplex assays and electrophoretic mobility shift assay (EMSA) analysis. Furthermore, blood samples were collected and immediately separated by centrifugation at 5000 rpm and stored at − 80 °C until assayed. Thalidomide (Fundação Ezequiel Dias-FUNED, Belo Horizonte, Brazil) was dissolved in dimethylsulfoxide (DMSO) (Sigma Chemical Co., Saint Louis, MO) solution, with a final DMSO concentration of 10%.

### Biochemical determinations

Blood samples were drawn from a pre-existing right carotid arterial cannula. Serum levels of alanine aminotransferase (ALT), aspartate aminotransferase (AST), lactate dehydrogenase (LDH), and alkaline phosphatase (ALP) were quantified using a Cobas c111 analyser (Roche, Indianapolis, IN) at the Liver Transplantation Laboratory of the University of Sao Paulo, according to standard procedures.

### Liver tissue immunohistochemical analysis

Macrophages (M1 and M2 cells) were detected in paraffin-embedded liver sections. Optimal working dilutions of the primary antibody were determined previously by titration experiments. After blocking steps with 0.3% hydrogen peroxide and nonfat milk, the following primary antibodies were used: anti-rat CD68 (Abcam, California, EUA; diluted 1:100) and anti-rat CD206 (Abcam, California, EUA; diluted 1:800) to identify M1 and M2 macrophages, respectively. Negative control experiments were performed by omitting incubation with the primary antibody. Sections mounted on glass slides coated with 6% silane were deparaffinized in xylene, rehydrated through graded ethanol and in deionized water in the final step, and then subjected to microwave irradiation in citrate buffer to enhance antigen retrieval.

Preincubation with 10% normal horse serum (Vector, Burlingame, USA) in tris-buffered saline and with avidin and biotin was carried out to reduce nonspecific staining and to block nonspecific binding to endogenous biotin. The incubations with the primary antibodies were carried out overnight at 4 °C in a humidified chamber. Sections were then incubated with rat adsorbed biotinylated anti-mouse IgGs (Vector, Burlingame, USA), followed by incubation with the streptavidin–biotin-alkaline phosphatase complex (Dako, Glostrup, Denmark), both at room temperature. The sections were incubated with a freshly prepared substrate consisting of naphthol and fast red dye (Sigma, Saint Louis), counterstained with Mayer’s haemalaum (Merck, Darmstadt, Germany) and covered with Kaiser’s glycerin gelatine (Merck). Quantitative analysis of M1- and M2-positive cells was carried out in a blinded fashion under × 200 microscopic magnification and expressed as cells/mm^2^.

### Real-time reverse transcriptase polymerase chain reaction

Expression of TNF-α, IL-1β, IL-6, IL-10, MHC Class I and Class II, and NF-κB in liver tissue was analysed by real-time PCR. Total RNA from liver tissue was extracted (at 4 °C using a tissue homogenizer) by guanidinium thiocyanate-chloroform (Invitrogen, Carlsbad, USA) and isolated according to the manufacturer’s protocol. RNA quantity and purity were measured using a NanoDrop 2000c spectrophotometer (Thermo Scientific, Wilmington, USA). cDNA synthesis was performed using M-MLV Reverse Transcriptase from 1 μl of total RNA according to the manufacturer's protocol (Promega, Madison, USA).

Analysis of mRNA expression by reverse transcription RT–PCR was carried out using standard protocols. The following RT–PCR cycle profile was used: 10 min at 95 °C, followed by 40 cycles of 15 s at 95 °C for denaturation, 20 s at 60 °C for combined annealing, and 10 s at 72 °C for extension. Real-time PCR was performed using custom primers (Invitrogen, Carlsbad, USA). The sequences of the primers used for real-time PCR are shown in Table 1.

**Table 1:** Primer sequences used for Real Time PCR assays.

Gene	Sense and antisense (5′–3′)	Product (pb)
TNF-α	5′ TGGCCCAGACCCTCACACTCA 3′	541
	5′ GGCTCAGCCACTCCAGCTGC 3′	
IL-1β	5′ CCTTGTGCAAGTGTCTGAAGCAGC 3′	248
	5′ GCCACAGCTTCTCCACAGCCA 3′	
IL-6	5′ CCGGAGAGGAGACTTCACAGAGGA 3′	71
	5′ AGCCTCCGACTTGTGAAGTGGTATA 3′	
IL-10	5′ TCAGTCACATTTGTTTTCTGCAAA 3′	65
	5′ CTGCAAAAGTGGAGCAGTCATT 3′	
MHC class I	5′ TTCCTGCTACCGTTCCTCAC 3′	65
	5′ GGTGTGAGTCCACATACCCA 3′	
MHC class II	5′ TCAGTCACATTTGTTTTCTGCAAA 3′	65
	5′ CTGCAAAAGTGGAGCAGTCATT 3′	
NF-κB	5′ ATCAAAGAGCTGGTGGAGGC 3′	188
	5′ GAAGGCTGCCTGGATCACTT 3′	
β-actin	5′ AGGAGTACGATGAGTCCGGCCC 3′	70
	5′ GCAGCTCAGTAACAGTCCGCCT 3′	

### Detection of cytokine levels by multiplex assay

Briefly, frozen liver tissues were lysed and homogenized using RIPA buffer with protease inhibitors, as described on the data sheet. Thereafter, the homogenate was centrifuged at 14,000 rpm at 4 °C. The supernatants were collected and stored at − 80 °C until assayed. Total protein concentration was measured by the Bradford method. Cytokine levels of TNF-α, IL-1β, IL-6, and IL-10 were measured in liver tissue and in serum by a commercial MILLIPLEX® MAP kit (Millipore Corporation, Billerica, USA) according to the manufacturer’s instructions.

### EMSA of NF-κB consensus oligonucleotides

Nuclear extracts of rat liver tissue were prepared as previously described^13,20^. EMSA for the detection of nuclear NF-κB was performed using the gel shift assay kit from Promega as previously described^21^. For competition experiments, NF-κB and TFIID (5′-GCAGAGCATATAAGGTGAGGTAGGA-3′) unlabelled double-stranded consensus oligonucleotides were included in a 2.5-fold molar excess over the amount of 32^P^-NFκB probe to detect specific and nonspecific DNA–protein interactions, respectively. Nuclear extracts of the liver tissue presented a similar pattern of DNA/protein complexes as previously reported^13^. The upper complexes were displaced by an excess of unlabelled NF-κB, demonstrating the specificity of the NF-κB/DNA binding interaction. Supershift assays using antibodies against different NF-κB subunits (p50, p65 and cRel, 1:20 dilution) were also conducted according to the manufacturer’s protocol (Santa Cruz Biotechnology, Santa Cruz, CA) before the incubation of nuclear extracts with the labelled oligonucleotide. Autoradiographs were visualized with a DP-001-FDC photo documentation system (Vilber, Lourmat, France) and quantified by NIH ImageJ software (http://rsb.info.nih.gov/ij). Several exposure times were analysed to ensure the linearity of the band intensities.

### Statistical analysis

Data are presented as the means ± SEM. The normality of the data distribution was checked through skewness, kurtosis statistics, and graphical methods. Statistical analyses were performed with Prism statistical software (GraphPad, San Diego, USA). One-way ANOVA with pairwise comparisons according to the Newman–Keuls formulation was used. *P*˂0.05 was considered significant.

## Results

### Clinical parameter analyses

**Figure 2:**
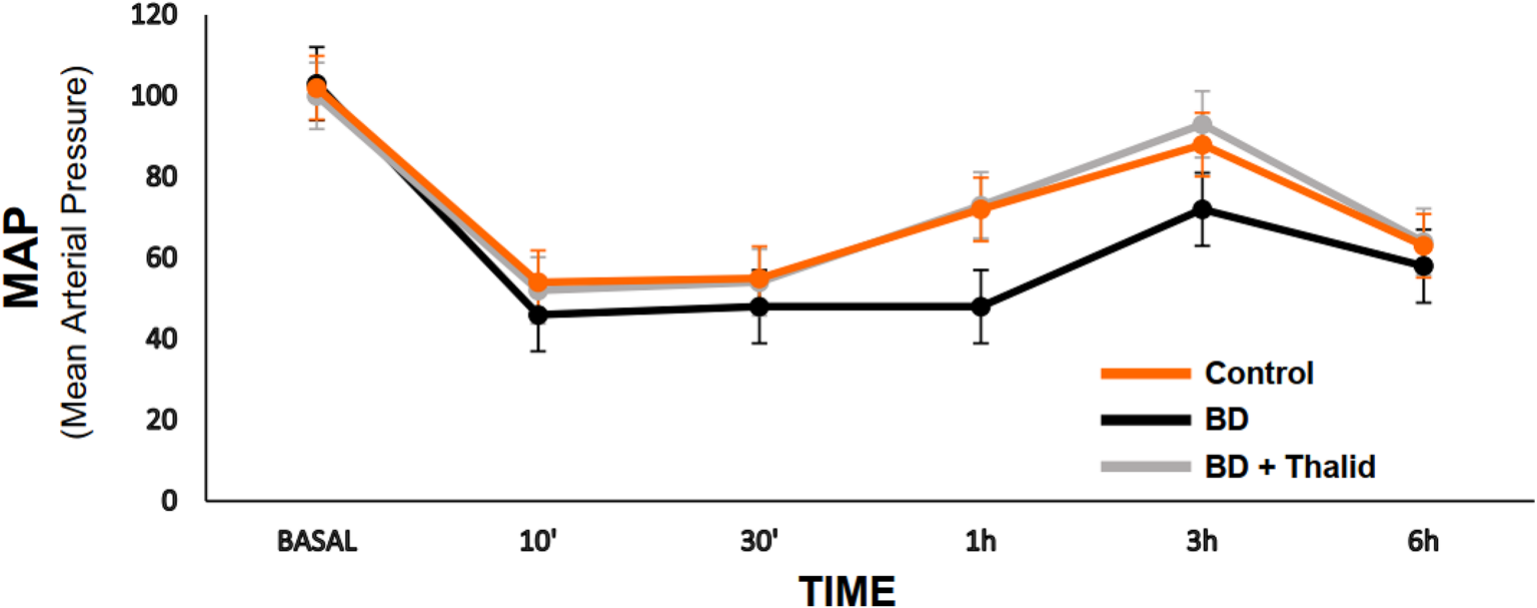
Mean arterial pressure analysis 6 h after brain death induction in the different groups.

The dose of thalidomide used in this study was well tolerated and did not affect the haemodynamic stability of the animals. The mean arterial pressure in all groups was maintained above 50 mmHg during the entire follow-up period of the experimental groups without the use of vasoactive drugs or colloids. Although the mean blood pressure was higher in the animals treated with thalidomide, no significant differences were found among the groups (Fig. 2).

### Effects of thalidomide on hepatic injury markers

Assessing hepatic injury, the serum levels of ALT, AST, LDH, and ALP were significantly higher in the BD group than the control group. These enzymes were reduced by thalidomide treatment (Table 2).

**Table 2:** The serum levels of ALT, AST, LDH, and ALP.

Parameters (IU/L)	Groups		
	Control	BD	BD + Thalid
ALT	66.6 ± 9.2	232.7 ± 40.9*	83.4 ± 5.2^#^
AST	140.1 ± 3.9	349.2 ± 26.5*	230.7 ± 32.2^#^
LDH	267.1 ± 17.7	411.1 ± 12.4*	244.9 ± 30.2^#^
ALP	99.2 ± 9.5	135.8 ± 13.2*	102.4 ± 8.7^#^

The samples were analysed by clinical chemistry testing. Data are expressed as the means ± SEM.

*p < 0.05 versus Control group.

^#^*p* < 0.05 versus BD group.

### Thalidomide diminishes macrophage infiltration in liver tissue

**Figure 3:**
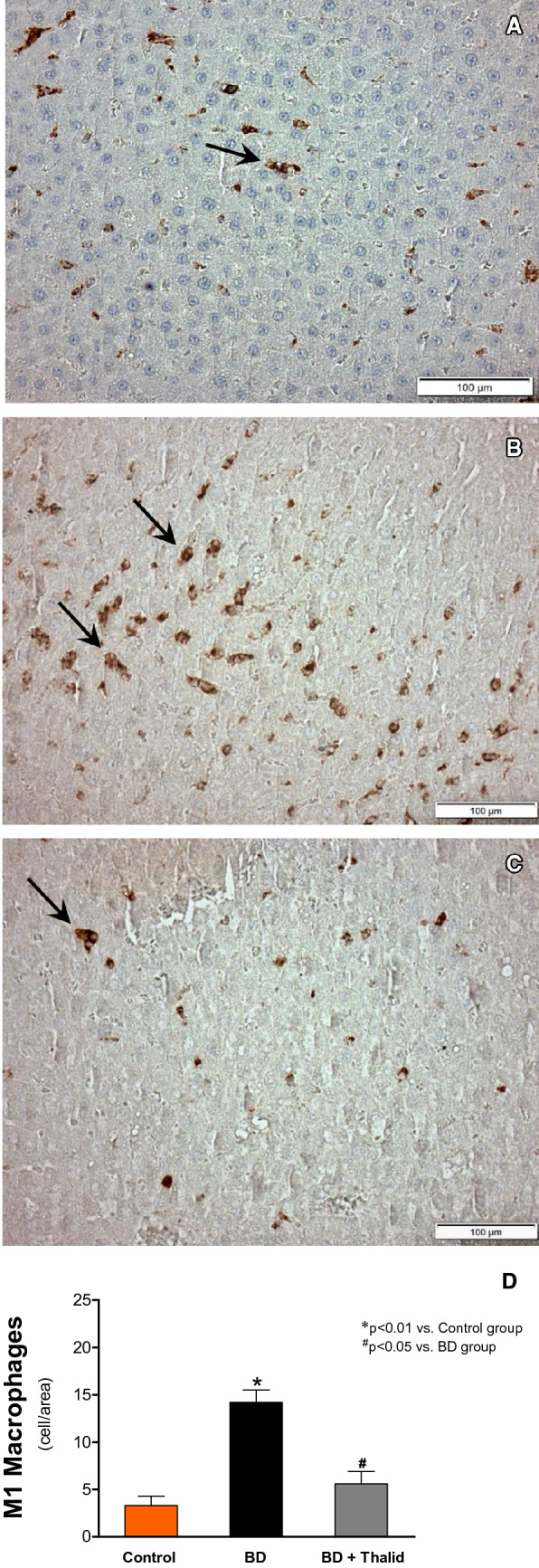
Immunohistochemical detection (**A**–**C**) (arrow) and comparative analysis (**D**) of M1 macrophages 6 h after brain death in the different groups. Original magnifications: × 400.

The anti-inflammatory effects of thalidomide on liver tissue were demonstrated by immunohistochemistry staining. In the BD group, the immunohistochemical assays demonstrated significant M1 macrophage infiltration compared with the control group (Fig. 3A, B). On the other hand, animals that received thalidomide treatment presented a significant reduction in the number of infiltrating macrophages in the liver tissue (Fig. 3C). No significant differences were detected in the mean number of infiltrating M2 macrophages. The M1 macrophage results are shown in Fig. 3D.

### Immunologic status of the liver is ameliorated by thalidomide

**Figure 4:**
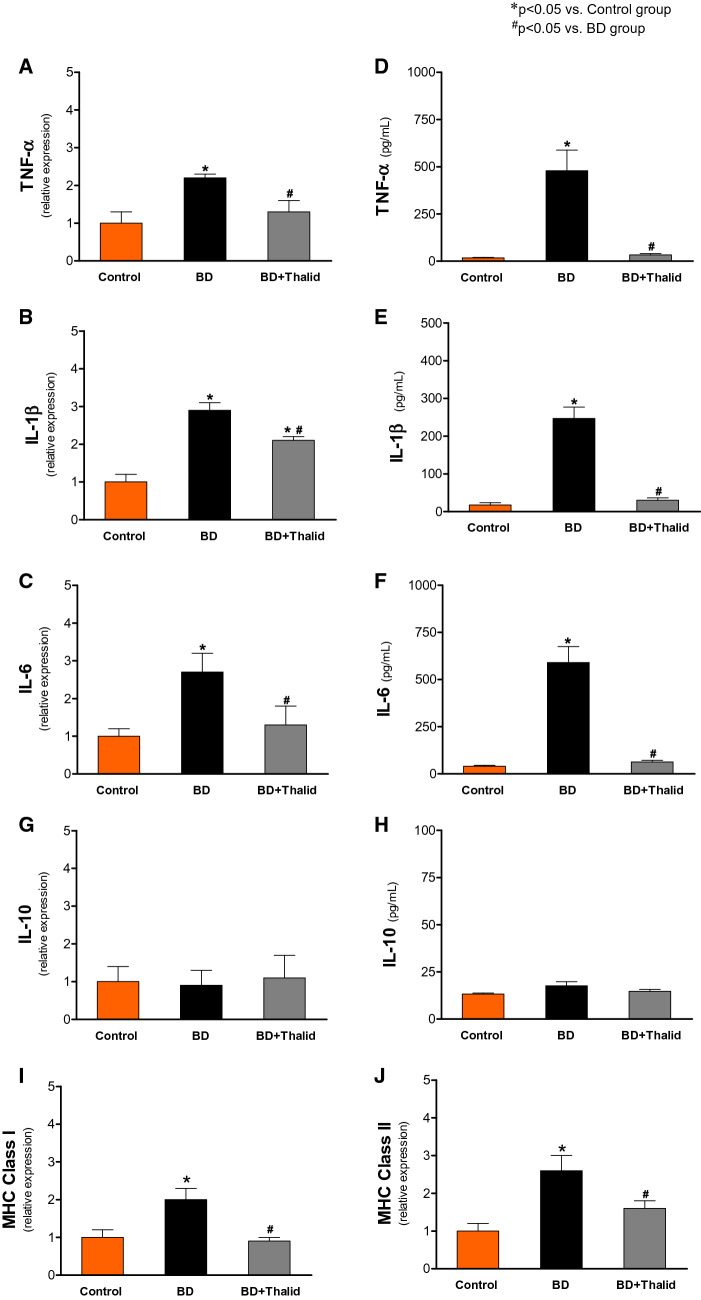
Comparative analysis of inflammatory cytokines 6 h after brain death in the different groups. The expression of TNF-α (**A**), IL-1β (**B**), IL-6 (**C**), IL-10 (**D**), (**I**) MHC Class I and (**J**) MHC Class II was analysed by real-time RT–PCR in liver tissue. The cytokine levels of TNF-α (**E**), IL-1β (**F**), IL-6 (**G**), and IL-10 (**H**) were measured using a MILLIPLEX/LUMINEX MAP kit.

To confirm the involvement of inflammatory mediators in the liver in this experimental model, the mRNA levels and tissue concentrations of inflammatory cytokines, such as TNF-α, IL-1β, IL-6, and IL-10, were analysed by real-time PCR and multiplex assays, respectively. In the BD group, the mRNA levels and concentrations of TNF-α, IL-1β, and IL-6 in liver tissue were significantly higher than those in the control group (Fig. 4A–F). These cytokines were significantly reduced by thalidomide treatment (Fig. 4A–F). In contrast, the analysis of IL-10 data showed no significant difference among the groups (Fig. 4G, H). Furthermore, in the animals of the BD group, the mRNA levels of MHC Class I and MHC Class II were significantly higher than those in the control group. Thalidomide also promoted a significant reduction in the expression of these genes (Fig. 4I, J).

### Effects of thalidomide on systemic inflammation

Signs of systemic inflammation were demonstrated in the donor BD model by the detection of remarkably high levels of serum cytokines TNF-α, IL-1β, and IL-6 in the BD group compared with the control group. In contrast, in animals that received thalidomide treatment, a decrease in these cytokines was observed, as shown in Table 3. There were no significant differences in IL-10 serum levels.

**Table 3:** The serum levels of TNF-α, IL-1β, IL-6, and IL-10.

Parameters (pg/mL)	Groups		
	Control	BD	BD + Thalid
TNF-α	2.1 ± 0.4	46.8 ± 10.4*	4.6 ± 0.9^#^
IL-1β	0.8 ± 0.4	27.9 ± 4.0*	4 ± 0.4^#^
IL-6	2966.5 ± 793.7	9310.0 ± 976.8*	3689.5 ± 634.6^#^
IL-10	307.7 ± 70.3	355.3 ± 94.6	121.8 ± 22.4

The samples were analysed by multiplex cytokine assay. Data are expressed as the mean ± SEM.

*p < 0.05 versus Control group.

^#^*p* < 0.05 versus BD group.

### Thalidomide inhibits NF-κB activation in liver tissue

**Figure 5:**
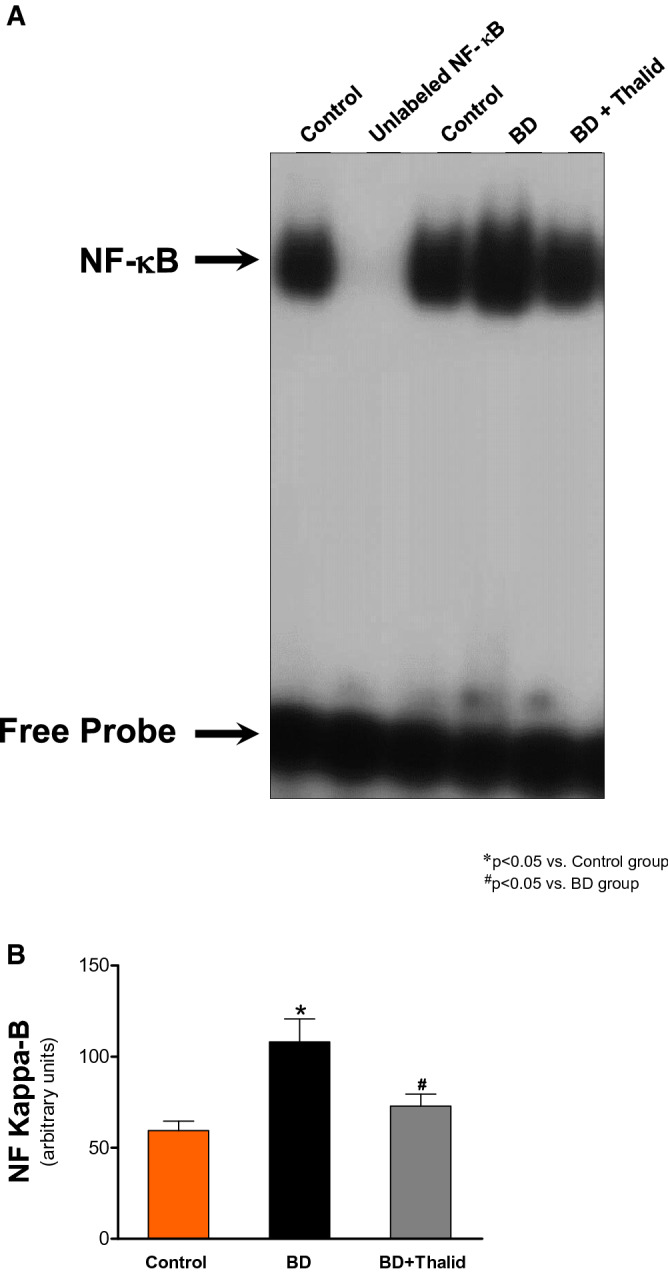
The results represent the effects of thalidomide on NF-κB activation in the liver in a rat donor brain death model. (**A**) Nuclear proteins were extracted from the liver of rats treated with thalidomide (BD + Thalid) (200 mg/kg), the control group and the brain death group (BD). Nuclear proteins (10 µg) were used to perform EMSA to evaluate NF-κB activity. Competition studies were performed using 10 µg of nuclear extract from the control group in the presence of 20-fold molar excess unlabelled specific NF-κB consensus sequence. (**B**) Densiometric analysis (arbitrary units, A.U.) of the NF-κB band is shown in Panel B. The composition of the specific NF-κB/DNA binding complex and the free probe is indicated. The results are expressed as the means ± SEM from 3 individual experiments. *p < 0.01 BD versus Control (n = 4); ^#^*p* < 0.05 BD + Thalid versus BD (n = 4) (one-way ANOVA followed by Newman–Keuls test). The raw pictures of the EMSA are shown in Supplementary Fig. S1.

**Figure 6:**
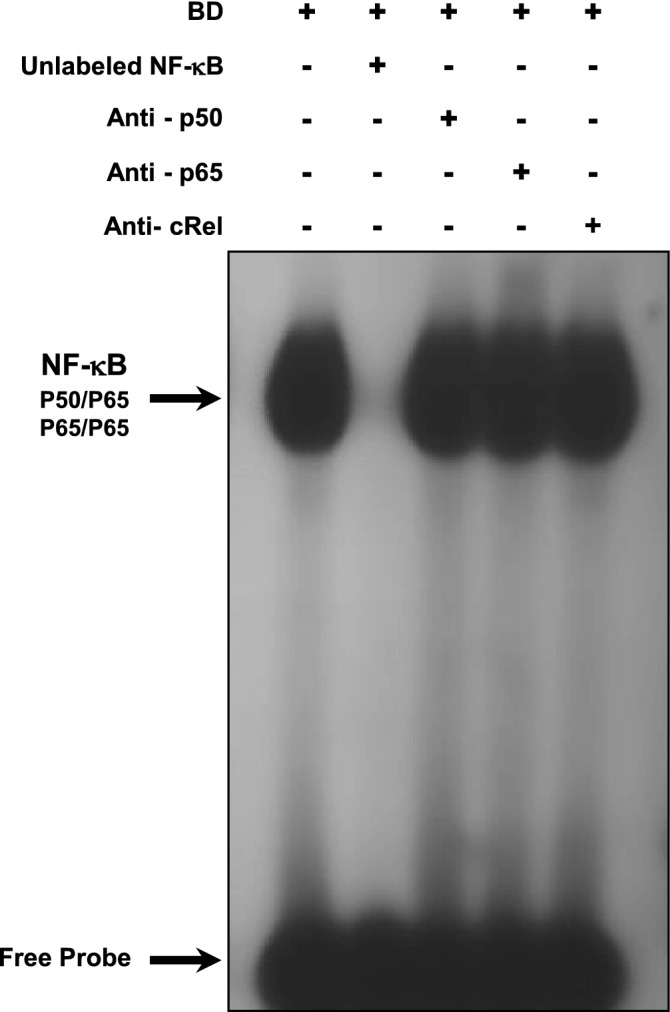
EMSA competition studies and supershift assays performed using nuclear extracts from hepatic tissue samples in the presence of specific oligonucleotides. Supershift assays were performed on nuclear extracts incubated in the absence and presence of antibodies against the p65, p50 and cRel subunits. The position of the specific NF-κB-binding complex (p50/p65) is indicated. The results are representative of three experiments. The raw pictures of the EMSA supershift assay are shown in Supplementary Fig. S2.

EMSAs were performed to investigate whether thalidomide blocks NF-κB binding activity in liver tissue induced by a donor BD model. EMSA for NF-κB revealed increased nuclear translocation of this transcription factor in the liver tissue of the BD group (Fig. 5A, B). Thalidomide treatment also significantly reduced activation of the transcription factor NF-κB in the liver tissue (Fig. 5A, B). The NF-κB complex was displaced by an excess of unlabelled NF-κB, demonstrating the specificity of the NF-κB/DNA binding interaction (Fig. 6). Supershift analysis indicated that the antibody against the p50 and p65 subunits induced a partial decrease in the NF-κB complex. The presence of antibodies against the c-Rel subunit did not affect the DNA–protein complexes (Fig. 6). Taken together, these results indicated that p50/p65 heterodimers and p65/p65 homodimers are likely included in NF-κB/DNA.

**Figure 7:**
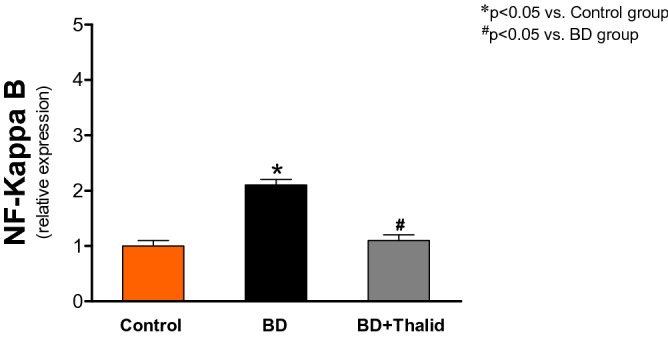
Comparative analysis of the expression of NF-κB as analysed by real-time RT–PCR in liver tissue 6 h after brain death induction in the different groups.

Furthermore, in animals of the BD group, the mRNA levels of NF-κB were significantly higher than those in the control group. Thalidomide also promoted a significant reduction in the expression of this gene (Fig. 7).

## Discussion

For the first time, animals subjected to BD were treated with thalidomide, a drug with powerful anti-inflammatory and immunomodulatory effects^22,23^. The present study used rats in a BD donor model to investigate the immunological mechanisms involved in this process. We evaluated the presence of inflammatory cell infiltration, expression of inflammatory cytokines, and NF-κB activation in the liver tissue.

Thalidomide continues to be associated with teratogenic effects that were devastating in the past. Doctors and researchers still have an irrational fear of using or studying the drug, even in populations out of reproductive age or in men, because of its effects on pregnant women. The proposed target population of the drug as suggested by this study would be brain death organ donors; consequently, the use of the drug would be in a medical institution under the care of an organ procurement organization (OPO). Medical practice is becoming more expensive every day, and the study and development of new drugs are long and costly. Society cannot deprive itself of existing drugs, testing and maximizing the use of all its benefits, with proven safety based on science. In this study, thalidomide was shown to be beneficial to the damage caused by brain death.

BD is followed by a generalized inflammatory response, leading to an increased inflammatory infiltrate and expression of cytokines at the intraorgan level. This condition might lead to increased cellular adhesion molecules, as well as MHC complexes on donor cells, increasing the immunogenicity of the organ and subsequently amplifying posttransplant rejection^24^. Indeed, clinical and experimental studies have documented graft survival inferiority of organs from BD donors compared with living donors, suggesting that BD is a risk factor for rejection in organ transplantation^25^. Therefore, interventions that seek to reduce the immunomodulatory response after BD may be relevant. Nonetheless, the underlying immunomodulatory mechanisms by which BD leads to these processes remain unclear.

In this context, the BD donor model using rats was shown to be a useful animal model that resembles the pathophysiological aspects of BD organ donors in humans^19,26^. Another advantage of this experimental model is the ability to investigate the effects of individual drugs without any unwanted drug interactions.

Serum sample analysis identified a heightened systemic inflammatory state characterized by circulating proinflammatory cytokines, including TNF-α, IL-1β, and IL-6. Although the mechanisms behind systemic inflammation during BD are not well understood, studies have shown that dysfunction of the blood–brain barrier and increased intestinal permeability release cytokines into the circulation^27,28^. The presence of increased plasma levels of proinflammatory cytokines has been recognized as an important risk factor for liver cell injury and cell death^29^. In addition, the sympathetic response with catecholamine release triggers a systemic inflammatory response by changing aerobic to anaerobic metabolism^30,31^.

Immunohistochemical experiments identified the remarkable presence of M1 cells in liver tissue, indicating that BD provokes an inflammatory response in peripheral organs^32^. The involvement of local inflammation in this model was further confirmed by a marked upregulation of inflammatory gene expression and tissue protein concentration observed in liver tissues of the BD group, including TNF-α, IL-1β, and IL-6. These findings are supported by previous results that showed upregulation of inflammatory cytokines in liver tissue, indicating that BD evokes an immune reaction in the liver^9,33^. This process is characterized by eventual hepatocyte necrosis, hepatic artery injury, biochemical abnormalities, some fibrosis, and therefore decreased graft survival. In addition, cytokines may also directly damage the liver tissue acutely, amplifying this response^34^. An important point must be raised, however. Nonparenchymal cells, such as Kupffer cells, liver sinusoidal endothelial cells, hepatic stellate cells, and lymphocytes, could influence these inflammatory processes through different pathways. Thus, in vitro studies could be useful to elucidate the specific role of these cells in the inflammatory response.

In this study, the anti-inflammatory and immunomodulatory effects of thalidomide were confirmed through suppression of local and systemic cytokines, particularly TNF-α, IL-1β, and IL-6. These findings are in agreement with other studies that showed that this drug has the ability to downregulate many cytokines^13,16,22,23^. For example, thalidomide enhances the degradation of TNF-α mRNA, thus reducing the release of this proinflammatory cytokine from endotoxin-stimulated macrophages^35^. In addition, thalidomide has a direct effect on nitric oxide (NO) by decreasing TNF-α, IL-1β and IFN-γ synthesis. These cytokines are important mediators of NO production because they regulate the expression of inducible nitric oxide synthase (iNOS)^36–38^. Proinflammatory cytokines also play a key role in the initiation and progression of hepatic disorders, as reflected in increased ALT, AST, LDH, and ALP levels, whereas thalidomide inhibited these effects.

In parallel, MHC class I and class II gene expression was elevated in liver tissues of the BD group, thereby influencing liver graft immunogenicity. MHC antigens are the most important alloantigens responsible for eliciting potent antigraft immune responses^37^. Briefly, T-cells recognize peptide antigens bound to MHC molecules through their T-cell receptor (TCR), and each T-cell expresses a unique TCR that binds to a particular MHC-peptide complex. Mature T-cells bear either CD4 or CD8 coreceptors, and these bind to nonpolymorphic regions of MHC class II and class I, respectively, on antigen-presenting cells, mechanisms that are responsible for graft rejection^37,39^. Several studies have also identified an important role of cytokines in modulating both MHC class I and class II antigens, combined with the expression of adhesion molecules^40–43^.

There is a great body of evidence that proinflammatory cytokines, particularly TNF-α, can upregulate MHC class I and class II expression in different cell lines, indicating that this cytokine is strongly associated with immunogenicity^42,44^. As a result, MHC antigens from donors are recognized by the recipient’s immune system, triggering an allogeneic immune response, which in turn provides essential signals for organ rejection mechanisms^45^. In this context, the beneficial downmodulation of immunogenicity by thalidomide may suppress this process in organ donor brain death, as well as the development of allorecognition after transplantation.

The activation of NF-κB was also found in the livers of the BD group. NF-κB has an important role in controlling the expression of genes that mediate immune responses and immunogenicity^46^. Additionally, there is an association between NF-κB and the regulation of MHC Class I expression^47,48^. On the other hand, inflammatory mediators, such as TNF-α and IL-1β, are able to activate NFκB, which may result in a positive-feedback loop in the liver, as discussed above^46^. These cytokines lead to activation of inhibitory κB proteins, which are mediated by the IKK complex. The IKK complex phosphorylates IκB, leading to degradation and allowing nuclear translocation of NF-κB complexes, e.g., Rel-A/p50 and c-Rel/p50^49^. In the nucleus, NF-κB binds to its cognate DNA binding sites to regulate the transcription of immunomodulatory genes^46^. In this context, our results also show that antibodies against the p50 and p65 subunits induced a partial decrease in the NF-κB complex, indicating that p50/p65 heterodimers and p65/p65 homodimers are likely included in NF-κB/DNA.

Although several aspects of this complex scenario related to the immunological response in BD organ donors have been recognized, few advances in the improvement and treatment of this condition have been achieved. Intervention therapies are of crucial relevance since no recognized treatment has been shown to be beneficial in BD organ donors with signs of inflammation.

Therefore, we postulated that thalidomide could mediate its effects through a reduction in NF-κB activation. Indeed, previous studies strongly indicated that thalidomide is a potent inhibitor of NF-κB activation^13,17,49,50^.

In the present study, thalidomide indeed inhibited NF-κB expression in liver tissue, confirming the involvement of this transcription factor in the mechanistic effects of this drug. It is important to stress that different processes of immune responses can activate NF-κB, particularly in response to TNF-α, and in cells in which NF-κB upregulates the expression of a large variety of genes, including TNF-α and MHC^48,49^. In that respect, NF-κB could also be considered an important target to regulate liver immunogenicity after brain death.

The harmful effect of inflammation in donor liver tissue was likely overcome by the immunomodulatory effects of thalidomide, which attenuated the progression of inflammatory mechanisms involved in this pathophysiological condition. Thalidomide possesses a broad range of biological effects on the modulation of cytokine production, leading to its classification as an immunomodulatory drug. Inhibition of TNF-α production has been shown to be one of the main mechanisms responsible for the effects of thalidomide, as demonstrated in a model of biliary cirrhosis^50^ and further confirmed through the blockade of systemic production of TNF-α in carotid artery neointimal hyperplasia^23^, adenine-induced chronic kidney disease^13^, and an experimental model of burn^22^.

In patients with erythema nodosum leprosum, thalidomide ameliorates systemic inflammatory symptoms and promotes a reduction in serum TNF-α levels^51,52^. Reduction of plasma levels of TNF-α associated with thalidomide treatment was also described in patients with tuberculosis, with or without HIV infection, with increased weight gain^53^. Although the mechanisms underlying the immunomodulatory effects of thalidomide have not been completely clarified, suppression of TNF-α production may be a result of increased degradation of mRNA induced by thalidomide^35^. Other studies have provided evidence of a broader effect of thalidomide on cytokine production associated with the blockade of NF-κB-regulated genes via the inhibition of IκB kinase activity^17,18^. Indeed, investigators have shown that NF-κB activity is regulated by signal-induced IκB degradation, leading to NF-κB activation^46,54^. In addition to inhibiting TNF-α, thalidomide has been shown to decrease the production of IL-1β and IL-6, in agreement with the findings of our study^55,56^.

Considering the evidence of the negative impact of the immunologic response induced by BD, the present study clearly demonstrates that thalidomide may represent an important strategy to improve the quality and survival of organs from brain-dead donors. Nevertheless, this is an exploratory study, and further studies are needed before translating these experimental findings into clinical applications. Moreover, despite its history as a human teratogen, thalidomide has shown impressive activity in various clinical and experimental disorders, including haematologic malignancies, inflammatory diseases, HIV, and cancer, among others^57–60^.

## Conclusion

The results of this study demonstrated that BD is a complex process evoking a systemic immune response as well as a local inflammatory response in liver tissue. The immunomodulatory properties of thalidomide were effective in decreasing systemic and local proinflammatory cytokines, as well as inflammatory cell infiltration, leading to an improvement in liver damage, as reflected in decreased hepatic enzyme levels. Furthermore, the immunomodulatory effects of thalidomide possibly occurred through suppression of NF-κB activation. In addition, these data show evidence of potent immunomodulatory effects of thalidomide that appear to prevent increased immunogenicity. Taken together, these data provide key insights into the immune response in brain-dead organ donors as well as the potential use of thalidomide as an immunosuppressant. Perhaps, thalidomide may become a target for future therapies directed towards ameliorating graft immunogenicity and beneficial outcomes after transplantation.

## Supplementary Information

Supplementary Information.
