# Supplementary material for: Immunomodulatory effects of thalidomide in an experimental brain death liver donor model
Source: Sci Rep. 2021 Sep 28;11:19221. doi: 10.1038/s41598-021-98538-z (PMC8479052; doi:10.1038/s41598-021-98538-z)
Supplement: Supplementary file 1 — Supplementary Information. [file 41598_2021_98538_MOESM1_ESM.pdf]

Immunomodulatory effects of thalidomide in a experimental brain death liver donor model

*Alexandre Chagas Santana[1,4], Wellington Andraus[2] Filipe Miranda Oliveira Silva[3], Humberto Dellê[3], Rafael Pepineli[3], Edvaldo Leal de Moraes[4], Cristoforo Scavone[5], Larissa de Sá Lima[5], Sabrina Degaspari[5], Sergio Brasil[1], Davi Jorge Fontoura Solla[1], Liliane de Moreira Ruiz[6], Karina Andrighetti Oliveira-Braga[6], Natalia Aparecida Nepomuceno[6], Paulo Manuel Pego-Fernandes[6], Stefan Gunther Tullius[7], Eberval Gadelha Figueiredo[1]

[1] Neurological Surgery Department, University of Sao Paulo School of Medicine, Sao Paulo, Brazil

[2] Gastroenterology Department, University of Sao Paulo, School of Medicine, Sao Paulo, Brazil

[3] Medical Science Department, Nove de Julho University, São Paulo, Brazil

[4] Organ Procurement Organization Department, University of Sao Paulo, School of Medicine, Sao Paulo, Brazil

[5] Molecular Neuropharmacology Laboratory, Department of Pharmacology, University of Sao Paulo, Institute of Biomedical Science, Sao Paulo, Brazil

[6] Cardiopneumology Department, University of Sao Paulo, School of Medicine, Sao Paulo, Brazil

[7] Department of Surgery, Division of Transplant Surgery, Brigham and Women's Hospital, Harvard Medical School, Boston, Massachusetts, USA

Address for correspondence
*Alexandre Chagas Santana
Neurological Surgery Department
University of São Paulo School of Medicine
Av. Dr. Enéas Carvalho de Aguiar, 255, 5th Floor,
CEP: 05402-000
São Paulo, Brazil
e-mail: alesantana@usp.br

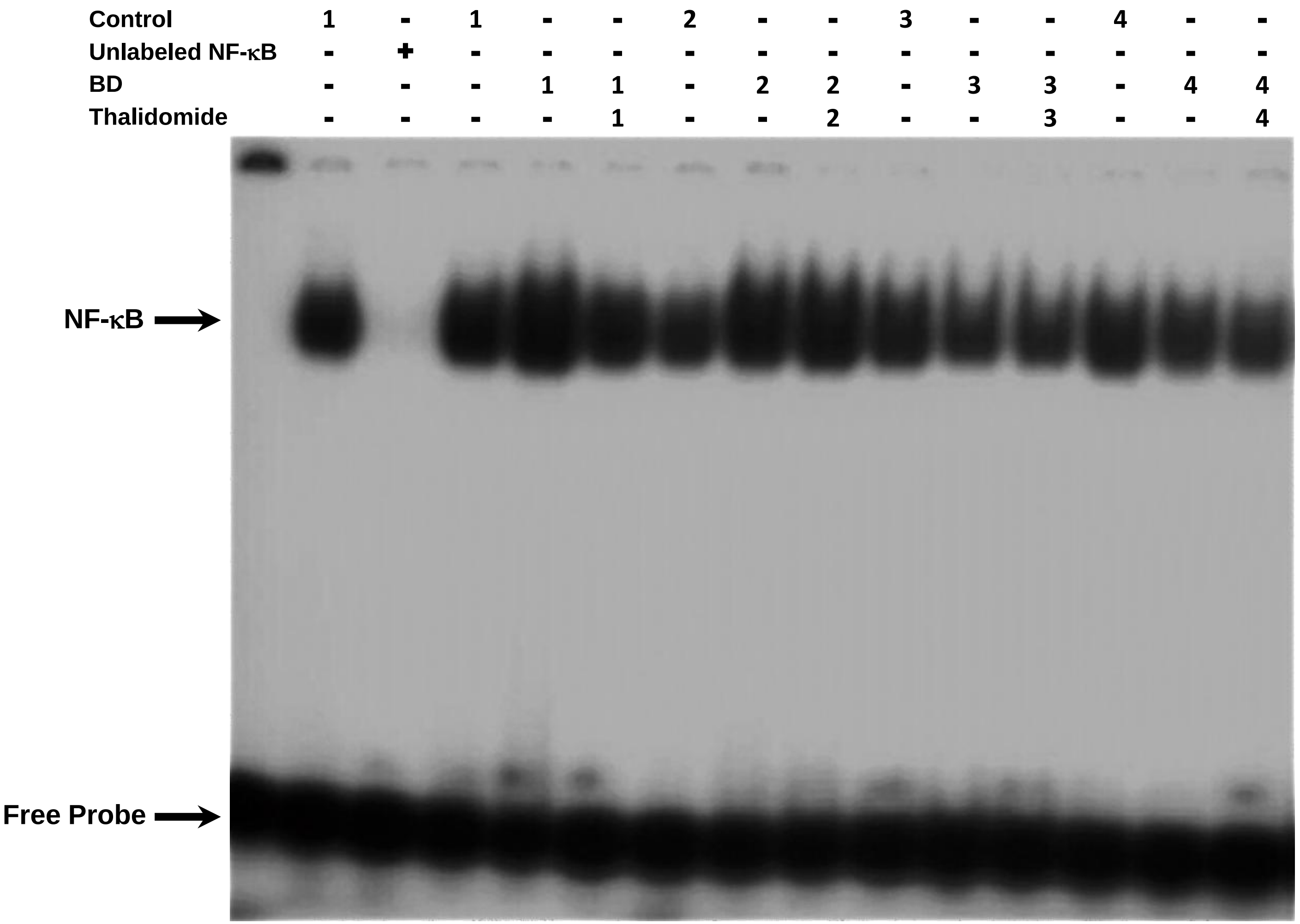

Supplementary Fig. S1: The results represents the effects of thalidomide in a donor brain death model on NF-κB activation in liver rats. Nuclear proteins were extracted from liver rats treated with thalidomide (BD + Thalid) (200mg/Kg); Control group and also brain death group (BD) (n=4 per group). 10μg of nuclear proteins were used to perform the EMSA assay to evaluated NF-κB activity. Competition studies were performed using 10μg of nuclear extract from control group in the presence of 20-fold molar excess unlabeled specific NF-κB consensus sequence. The composition of specific NF-κB/DNA binding complex and the free probe are indicated.

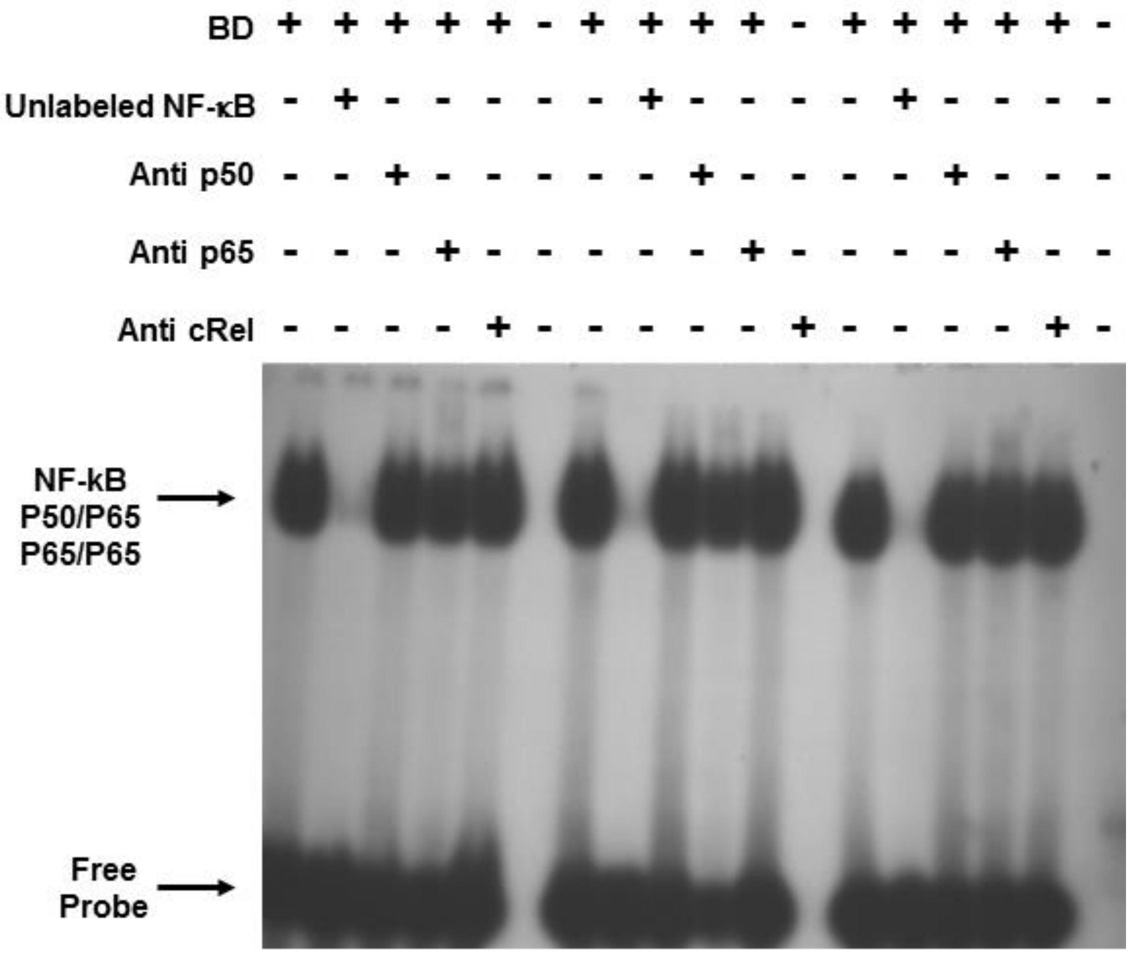

Supplementary Fig. S2: EMSA competition studies and supershift assays performed using nuclear extracts from hepatic tissue samples in the presence of specific oligonucleotide. Supershift assays were performed on nuclear extracts incubated in the absence and presence of antibodies against p65, p50 and cRel subunits. The position of specific NF-κB-binding complex (p50/p65) is indicated.
